# Occurrence, Risk, and Source of Heavy Metals in Lake Water Columns and Sediment Cores in Jianghan Plain, Central China

**DOI:** 10.3390/ijerph20043676

**Published:** 2023-02-19

**Authors:** Cong Wang, Kan Wang, Wuquan Zhou, Yong Li, Guoqing Zou, Zhi Wang

**Affiliations:** 1 Key Laboratory for Environment and Disaster Monitoring and Evaluation of Hubei, Innovation Academy for Precision Measurement Science and Technology, Chinese Academy of Sciences, Wuhan 430077, China; 2 University of Chinese Academy of Sciences, Beijing 100049, China; 3 Central-Southern Safety & Environment Technology Institute Co., Ltd., Wuhan 430051, China; 4 China Metallurgical Geology Bureau (CMGB) Bureau-1 (Hebei) Analysis & Technology Co., Ltd., Langfang 065201, China

**Keywords:** heavy metals, water column, sediment core, risk assessment, source apportionment

## Abstract

Heavy metal pollution in lakes is an issue that endangers ecosystems worldwide; however, the vertical properties of heavy metals in the water columns and sediment cores of lakes have been rarely evaluated simultaneously. This study revealed the pollution, risks, and sources of heavy metals from surface water to deep sediments in four typical shallow lakes located in central China. The results showed that the concentrations of heavy metals, except Hg, had insignificant stratification in the water column. Heavy metals had three vertical profiles in sediment cores, i.e., the concentrations of As, Hg, Cd, Pb, and Mn in the surface sediment (0–9 cm) were higher than that in the bottom sediment (9–45 cm) (*p* < 0.05), the concentrations of Cr, Co, Fe, and Ni in the bottom sediment were higher than the surface sediment (*p* < 0.05), and the concentrations of Cu and Zn had no significant stratification. The Nemerow pollution index showed that heavy metal pollution dominated by Hg reached slight–moderate levels, and had higher levels in surface water than that in bottom water (*p* < 0.05). The Nemerow integrated risk index showed that the heavy metals had moderate–extreme potential ecological risks (Cd contributed 43.4%) in the sediments, and the ecological risk in surface sediment was significantly higher than that in bottom sediment (*p* < 0.01). Principal component analysis revealed that agriculture, transportation, and chemical industry were the major sources of heavy metals in water and surface sediments, while agriculture and steel-making were the primary sources in bottom sediments. This study provides valuable data and insight for the control of heavy metal pollution in lakes with high human activity loads.

## 1. Introduction

Heavy metal pollution has become a problem due to its potential toxicity and persistence worldwide [1]. The pollution of heavy metals in the environment has reached a higher level than ever before due to anthropocentric activity (such as fossil fuel and mineral exploitation) in recent decades [1], although heavy metals widely existed in the natural environment before human use [2]. At present, freshwater lakes have become an important environment with heavy metal pollution [3]. Heavy metals in water can not only diffuse into organisms directly through drinking water, but also accumulate in aquatic organisms and spread in the biosphere via the food web; these phenomena could bring potential ecological and healthy risks [4,5]. Actually, heavy metals are difficult to degrade under natural conditions [6]. Adsorption and precipitation are important mechanisms for attenuating heavy metals in the water column [7]; thus, sediments become the source of heavy metals in the lake [8]. However, heavy metals have the opportunity for resuspension when environmental conditions (e.g., pH and temperature) change in shallow lakes, and cause secondary pollution of the aquatic environment [9].

With rapid urbanization and industrialization, heavy metal pollution has become a common phenomenon in lakes in China [3]. Heavy metal pollution has been widely investigated and reviewed in China in the last decades [3,10,11], and is ubiquitous in the lakes of China at present, whether it is Taihu Lake on the plain [7] or Dianchi Lake on the plateau [12]. Jianghan Plain, as an alluvial plain of the Yangtze River and the Han River, is one of the most densely populated areas in China, and has hundreds of lakes [13]. Honghu Lake, Liangzi Lake, Daye Lake, and East Lake (Wuhan) are four typical shallow lakes, with an average depth of 1.5 to 3 m. These lakes provide water for aquaculture, irrigation, industry, and other human activities [14,15,16].

In recent years, the water quality of these lakes has rapidly deteriorated due to heavy metal pollution [16,17,18,19]. However, previous studies have focused on the concentration and risk of heavy metals in surface sediments, while ignoring the concentration of heavy metals along the vertical sediment profile. This research gap limits our understanding of the vertical distribution properties of heavy metals in the aquatic environment. Therefore, we can provide specific and scientific insights for the treatment of heavy metal pollution by assessing the pollution, risk, and source of heavy metals in the vertical profiles of the water columns and sediment cores.

In this study, 11 target heavy metals (As, Hg, Fe, Cr, Co, Ni, Cu, Zn, Cd, Pb, and Mn) were investigated in the water columns and sediment cores for the four lakes. The purpose of this study is as follows: (1) reveal the vertical distribution of heavy metals in the water columns and sediment cores; (2) evaluate the pollution level and ecological risks of heavy metals in the water columns and sediment cores; and (3) identify the pollution sources of heavy metals in the water columns and sediment cores. These analyses and assessments can provide more accurate and environmental schemes for the treatment of heavy metal pollution in lakes.

## 2. Materials and Methods

### 2.1. Study Area and Sample Collection

**Figure 1:**
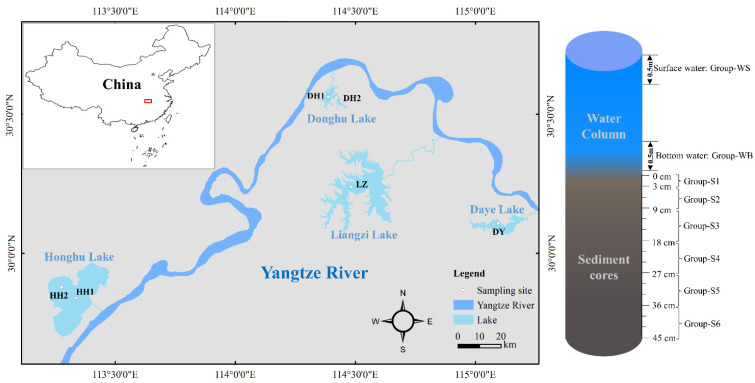
Spatial distribution and vertical profile of sampling sites. The red box represents the study area.

Honghu Lake, Liangzi Lake, Daye Lake, and East Lake have areas of 350, 300, 65, and 33 km^2^, respectively [14,15,16]. The four lakes are located in the Jianghan Plain (113°13′–115°12′ E, 29°40′–30°36′ N; Figure 1), and connected with the middle reaches of Yangtze River, Hubei Province, China. The subtropical monsoon climate is the common climate of the four lakes, and the annual average precipitation (1100–1300 mm) and temperature (15–17 °C) are similar. Superior habitation conditions facilitated human development and utilization of natural resources, and developed for agriculture and industry. For example, this province has the highest production of freshwater aquaculture and is in the top 10 for livestock production in China [13]. However, intense human activity has also led to the deterioration of the lake environment, including heavy metal pollution.

The sample collection campaign was conducted in the four lakes from 23 September to 26 September 2021. The sample sites include HH1, HH2, DH1, DH2, DY, and LZ (Figure 1). Water samples were collected 0.5 m from the surface and bottom. The sediments were collected via cylindrical corer with a diameter of 10 cm and cut at an interval of 3 cm for each sediment core. The water and sediment samples were stored in a refrigerator at −20 °C, and the pretreatment was completed within 48 h. Sites HH1, HH2, DH1, and DH2 had a 45 cm sediment core, and the sediment cores from Sites LZ and DY were only 27 cm because a hard clay layer was reached at a depth of ~20 cm.

### 2.2. Sample Analysis

The physiochemical parameters were determined for each sample. The water temperature (WT), dissolved oxygen (DO), electrical conductivity (EC), oxidation reduction potential (ORP), turbidity (Tur), chlorophyll-a (Chl-a), and pH of the water were measured in situ by using an EXO2 (YSI, Yellow Springs, OH, USA), and the ORP and pH of sediment cores were measured in situ with a portable detector. The total nitrogen (TN), total phosphorus (TP), ammonia nitrogen (NH_4_^+^-N), nitrate nitrogen (NO_3_^−^-N), nitrite nitrogen (NO_2_^−^-N), and orthophosphate (PO_4_^3−^-P) of water, and TN, TP, NH_4_^+^-N, NO_3_^−^-N, water content (WC), and organic matter (OM) of sediment were measured by spectrophotometer (for nutrients) and muffle furnace (for OM) based on the standard method [20,21]. The chemical oxygen demand was measured using the potassium permanganate (COD_Mn_) method.

Eleven heavy metals (As, Hg, Fe, Cr, Co, Ni, Cu, Zn, Cd, Pb, and Mn) were measured in the samples of surface water, bottom water, and sediment. Sediment cores were regrouped based on physiochemical parameters (Figure 1), and heavy metals were determined after being regrouped and mixed. The pretreatment and analysis of water and sediment samples were based on a previous study [12]. Briefly, 45 mL water sample, 4 mL HNO_3_, and 1 mL HCl were placed in a 100 mL closed Teflon vessel and digested 10 min at 170 °C; 0.1 g sediment sample and 6 mL aqua regia were put into a 100 mL closed Teflon vessel and digested 60 min at 180 °C. After digestion, the heavy metals Hg and As were determined by atomic fluorescence; Zn, Pb, Cu, Mn, Ni, Cd, Cr, Co, and Fe were determined by an inductively coupled plasma emission spectrometer [12].

### 2.3. Pollution and Risk Assessment

The single factor pollution index ($P_{i}$) was used to evaluate the pollution degree of specific heavy metals in water. The formula is as follows [22]:

(1) $$P_{i}=\frac{C_{i}}{S_{i}}$$

where $C_{i}$ is the measured concentration of heavy metal i, mg/L, and $S_{i}$ is the reference concentration based on the environmental quality standard of China [23] (Table S3). This standard only provides reference values for Cu, Zn, As, Hg, Cd, Pb, and Cr, so the remaining elements, Fe, Co, Mn, and Ni, were excluded from Equation (1). The pollution level of $P_{i}$ is listed in Table 1.

The Nemerow pollution index was performed to evaluate the comprehensive pollution level of heavy metals in water, and its calculation formula is as follows [24]:

(2) $$P_{n}=\sqrt{\frac{\max\left(P_{i}^{2}\right)+{\left(\bar{P_{i}}\right)}^{2}}{2},}$$

where $\max\left(P_{i}\right)$ is the maximum value of $P_{i}$ of heavy metals and $\bar{P_{i}}$ is the average value of $P_{i}$ of heavy metals. The pollution level of $P_{n}$ is shown in Table 1.

Additionally, the index of geoaccumulation ($I_{geo}$) was used to assess the pollution level of heavy metals in sediments, and the formula is as follows [25]:

(3) $$I_{geo}=Log_{2}\left(\frac{C_{i}}{1.5\times B_{i}}\right),$$

where $C_{i}$ is the measured concentration of heavy metal i and $B_{i}$ is the geochemical background value of heavy metal i (Table S4). The pollution level of $I_{geo}$ is listed in Table 1.

Recently, a new method, named the Nemerow integrated risk index (NIRI), was proposed to assess the potential ecological risks of heavy metals. This method not only considers the toxic reaction of heavy metals, but also eliminates the impact of the amount of heavy metals on the cumulative risk [26]. The equations of NIRI are as follows:

(4) $$NIRI=\sqrt{\frac{{\left(E_{r\;max}^{i}\right)}^{2}+{\left(E_{r\;ave}^{i}\right)}^{2}}{2}},\;$$

(5) $$E_{r}^{i}=T_{r}^{i}\times \frac{C_{i}}{S_{i}},\;\;$$

where $E_{r}^{i}$ is the potential ecological risk of heavy metal i [22]; $E_{r\;max}^{i}$ and $E_{r\;ave}^{i}$ are the maximum and average of $E_{r}^{i}$, respectively; $T_{r}^{i}$ is the toxicity coefficient of heavy metal i, and was obtained from previous study [27]; $\;C_{i}$ is the actual concentration of heavy metal i; and $S_{i}$ is the environmental background value of heavy metal i (Table S4). The risk rank of $E_{r}^{i}$ and NIRI is listed in Table 1.

### 2.4. Statistical Analysis

Pearson correlation is used to determine the change trend of physiochemical parameters with depth. Principal coordinate analysis (PCoA) based on Bray–Curtis distance and permutational multivariate analysis of variance (PERMANOVA) were performed to classify the concentration difference of heavy metal at different depths or in different lakes. The Wilcox test was used to compare the difference of concentration, pollution index, and risk of heavy metals at different depths or in different lakes. Principal component analysis (PCA) was used to explore the potential homology of heavy metals and physiochemical parameters. Statistical results are regarded as significant when *p* < 0.05. Data analysis and visualization were performed with R 4.2.1 (Revolution Analytics, Mountain View, CA, USA) and Origin Pro 2022 (OriginLab, Northampton, MA, USA).

## 3. Results and Discussion

### 3.1. Physicochemical Parameters in Water and Sediment

Physiochemical parameters of surface water and bottom water were similar (Table S1). Specifically, both surface water and bottom water were weakly alkaline (8.18 < pH < 9.27). The average values of WT and DO were 30.14 °C and 9.51 mg/L, respectively, during the sampling period. The range of EC, Chl-a, and COD_Mn_ was 186.0 to 414.0 μS/cm (mean 295.7 μS/cm), 8.37 to 29.46 µg/L (mean 17.02 µg/L), and 13.08 to 23.08 mg/L (mean 19.64 mg/L), respectively. The average concentrations of TN and TP were 0.87 and 0.14 mg/L, respectively. The physiochemical parameters showed that the four lakes we investigated have eutrophication and pollution to some extent [5]. 

As shown in Table S2, the sediment showed reducibility (the average values of pH and ORP were 6.39 and −216.4 mv, respectively), and the reducibility enhanced significantly with depth (pH and ORP decreased significantly with depth, *p* < 0.01). OM (5.9–14.6%) also had a significant increase tendency with depth (*p* < 0.01). For nutrients, the average concentrations of TN and TP were 2592.2 and 442.9 μg/g, respectively. The concentration of TN decreased significantly with increasing depth (*p* < 0.01); while TP had an insignificant change (*p* > 0.05). According to the US Environmental Protection Agency, sediment is considered seriously polluted when the concentration of TN and TP is more than 2000 and 650 μg/g, respectively [28]. These results suggested that the sediment cores of the lakes had serious TN pollution. The land adjacent to the four lakes has been cultivated for agriculture and aquaculture for a long time [14,15,16]; thereby, the ecological environment has been deteriorating continuously.

### 3.2. Concentration of Heavy Metals

#### 3.2.1. Heavy Metals in Water Columns

**Figure 2:**
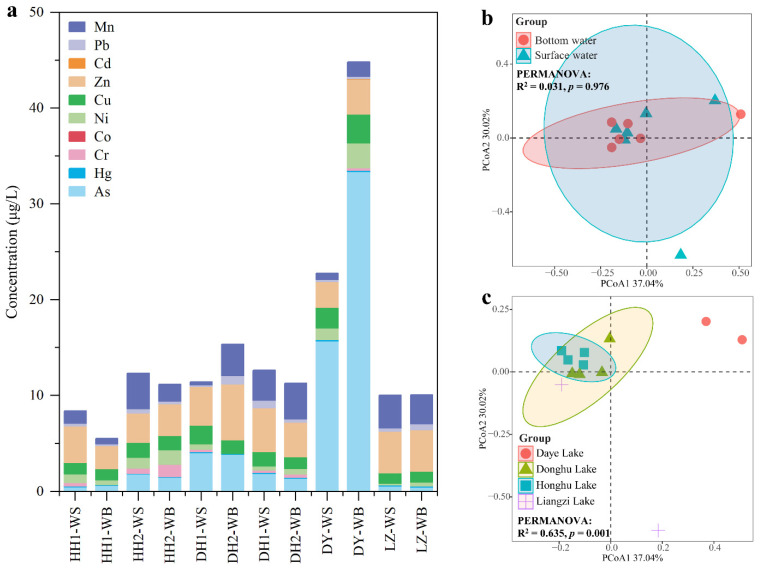
Heavy metals in the water columns: (**a**) concentration of heavy metals in surface water and bottom water; (**b**) distribution of heavy metals in surface water and bottom water; and (**c**) distribution of heavy metals in the water columns of different lakes. Oval represents 95% confidence interval.

Ten target heavy metals were detected in water (except for Co), including As, Hg, Ni, Cu, Zn, Pb, Mn, Fe, Cr, and Cd (Table S5). The total concentration of heavy metals ranged from 5.50 to 56.54 μg/L, and the order of average concentration was as follows: As (5.47 μg/L) > Fe (3.88 μg/L) > Zn (3.79 μg/L) > Mn (2.20 μg/L) > Cu (1.57 μg/L) > Ni (0.84 μg/L) > Mn (0.40 μg/L) > Cr (5.47 μg/L) > Hg (0.10 μg/L) > Cd (0.01 μg/L) (Figure 2a). The heavy metals in Daye Lake presented higher concentrations than the three lakes because of the extremely high As level (Figure 2a). Heavy metals showed no significant difference between surface water and bottom water based on PCoA and PERMANOVA (R^2^ = 0.031, *p* = 0.976) (Figure 2b), while a significant difference was recorded for Daye Lake and the other three lakes (PCoA and PERMANOVA: R^2^ = 0.635, *p* = 0.001; Figure 2c). These results indicate that the difference in heavy metals in different lakes is greater than that in the water column. 

The reported concentrations of typical heavy metals in the four lakes were compared with those in other surface waters in China (Table 2). The results showed that only As in Daye Lake was at the highest level when compared with lakes (Dianchi Lake, Taihu Lake, Chaohu Lake, Dongting Lake, and Caohai Lake) and rivers (Yangtze River, Haihe River, and Pearl River) in China. The concentrations of Hg, Ni, and Cu in the four investigated lakes were at middle or low level, while Mn, Pb, Cd, Zn, and Cr were at low rank (Table 2). Runoff input is regarded as an important source for heavy metals in water [29]. Thus, the discrepancy in the concentration of heavy metals in the different lakes possibly was affected by local industry. For example, the high As pollution in Daye Lake is linked with local metal smelting and mineral mining, which usually discharged untreated liquid and solid wastes [10].

#### 3.2.2. Heavy Metals in Sediment Cores

**Figure 3:**
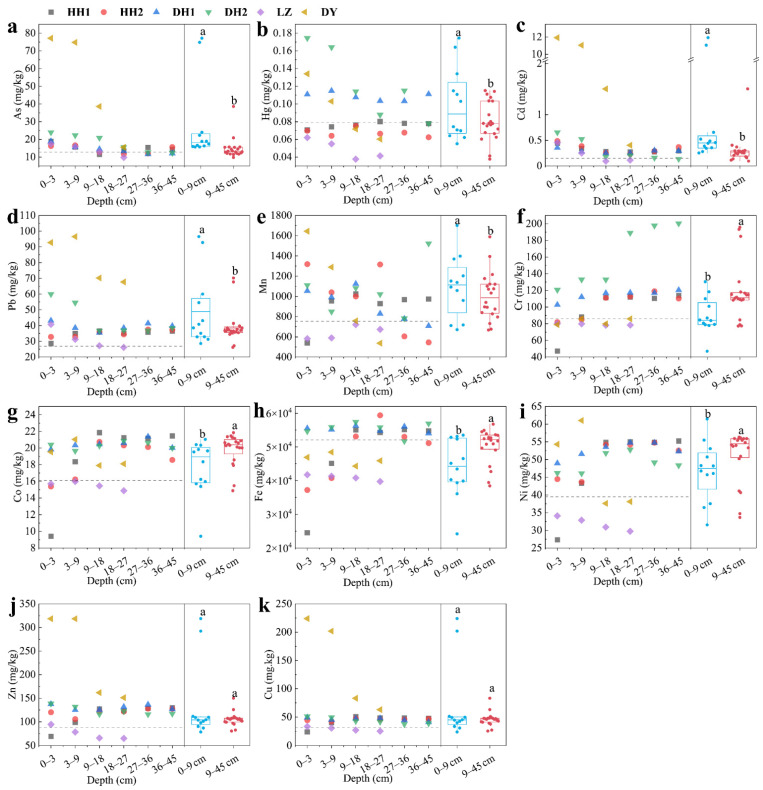
Concentration of target heavy metals in sediment cores (dry weight). Different letters (a or b) represent statistically significant differences at the *p* < 0.05 level. The gray dotted line is the environmental background value.

Eleven target heavy metals were all detected in the sediment samples (Table S6). The order of average concentration of heavy metals was: Fe (49,889.29 mg/kg) > Mn (932.98 mg/kg) > Zn (130.69 mg/kg) > Cr (109.70 mg/kg) > Cu (54.99 mg/kg) > Ni (47.60 mg/kg) > Pb (42.87 mg/kg) > As (19.71 mg/kg) > Co (19.00 mg/kg) > Cd (1.04 mg/kg) > Hg (0.09 mg/kg) (Figure 3). The average concentrations of 10 heavy metals (except Fe) were at higher levels when compared with the environmental background values reported for Hubei Province (Figure 3a–k). In particular, the average concentrations of Cd, Cu, Pb, and As were 6.9, 1.7, 1.6, and 1.5 times the background concentrations, respectively. The results imply that the four heavy metals were highly enriched. Similarly, Cd, Cu, Pb, and As in the adjacent Han River sediments also had high pollution levels [33]. These results suggest that Cd, Cu, Pb, and As could be considered as evidence for anthropogenic heavy metals in the Jianghan Plain and that the ecosystems of the four lakes have been strongly disturbed by human activity.

Heavy metals presented three vertical profiles in sediment cores (Figure 3). The first was that the concentrations of heavy metals (As, Hg, Cd, Pb, and Mn) in surface sediment (0–9 cm) were significantly higher than that in bottom sediment (9–45 cm) (Figure 3a–e); the second was that the concentrations of heavy metals (Cr, Co, Fe, and Ni) in bottom sediment had higher levels than those in the surface sediment (Figure 3f–i); and the third was that the concentration of heavy metals (Cu and Zn) had insignificant variation with the depth (Figure 3j–k). The different vertical profiles of heavy metals in the sediment cores can be attributed to different periods of human activity. The 45 cm deep sediment cores may cover sedimentary records with ~60 years in these lakes, based on previous investigations [34,35,36]. According to the deposition rate of sediments, we can speculate that humans have discharged more As, Hg, Cd, Pb, and Mn in the last decade, while producing more Cr, Co, Fe, and Ni pollution a few decades ago. In fact, As, Hg, Cd, Pb, and Mn are closely related to agricultural and industrial pollution [37,38], while fuel combustion and steel-making are considered as the primary source of Cr, Co, Fe, and Ni [39,40]. The inconsistent heavy metal pollution in different periods also corresponds to the industrial upgrading process of Jianghan Plain in the last half century, that is, the transformation from heavy industry to chemical and electric industry, and from traditional agriculture to modern agriculture [41]. Similarly, Cd, Hg, Pb, As, and Cr in Poyang Lake had the same vertical profiles with the four lakes we surveyed [42], which further confirms the impact of China’s industrial structure change on heavy metal pollution during the past half century.

The spatial distribution of heavy metals in the sediment was analyzed. The concentrations of As, Cd, Zn, Cu, and, Pb in Daye Lake (Site DY) were significantly higher than those in the other three lakes (*p* < 0.05), and Cr in East Lake (Site DH1 and DH2) had the highest concentration among these lakes (*p* < 0.05), while the concentrations of Hg, Fe, Co, Ni, Zn, and Mn in Liangzi Lake were significantly lower than those in other lakes (*p* < 0.05) (Figure S1). The reported concentrations of heavy metals in the four lakes we investigated were also compared with those in other sediments in China (Table 3). Ni, Mn, and Co had relative high levels in the four lakes when compared with the sediment in these lakes and rivers, while Hg, Fe, and Zn were at middle levels (Table 3). Additionally, As, Cu, Cd, and Pb in Daye Lake and Cr in East Lake presented higher levels than other lakes and rivers (Table 3). In fact, the concentration difference in heavy metals in sediments from different regions is affected by both the environmental background content of elements and the industrial structure [26]. For instance, the severe heavy metal pollution (e.g., As, Cu, Cd, and Pb) in Daye Lake is related to metal mining and smelting [10], while the Cr with high concentration in East Lake may be caused by the large amount of vehicle exhaust emissions [43]; East Lake is located in Wuhan (more than 10 million residents), the largest city in central China [13].

### 3.3. Pollution Assessment of Heavy Metals

**Figure 4:**
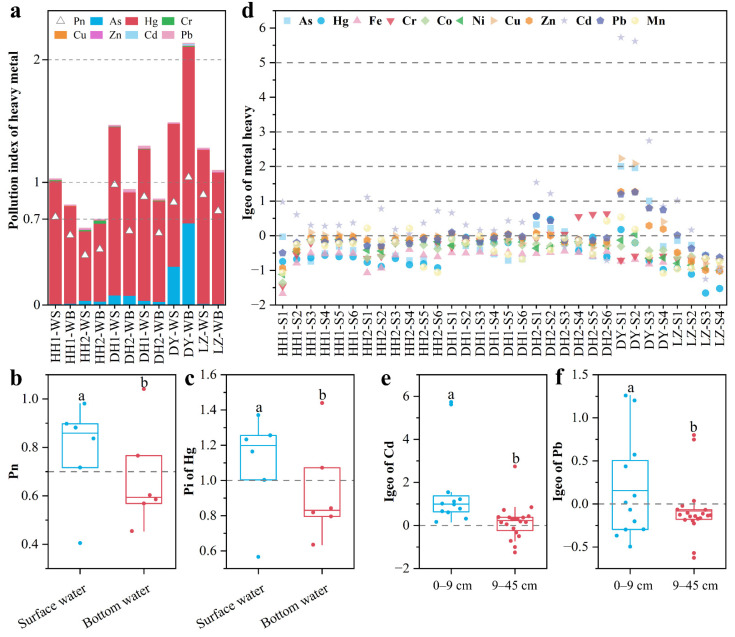
Pollution level of heavy metals: (**a**) pollution level of heavy metals in water columns; difference in $P_{n}$ (**b**) and $P_{i}$ for Hg (**c**) between surface water and bottom water; (**d**) pollution level of heavy metals in sediment cores and the difference in $I_{geo}$ for Cd (**e**) and Pb (**f**) within sediments at 0–9 and 9–45 cm. Different letters (a or b) represent statistically significant differences at the *p* < 0.05 level. The gray dotted line represents the pollution threshold of heavy metals: 1 and 2 represent slight and moderate pollution levels of specific heavy metal (*P_i_*) in water, respectively; 0.7 represent slight pollution level of all heavy metals (*P_n_*) in water; 0, 1, 2, 3 and 5 represent slight, moderate, moderate to heavy, heavy, and extreme pollution levels of heavy metals in sediments, respectively.

The comprehensive pollution index showed that heavy metals produced slight–moderate pollution in the water columns ($P_{n}$ > 0.7; Figure 4a). The single factor pollution index showed that water columns were slightly polluted by Hg ($P_{i}$ > 1), and that Hg was the primary contributor (mean 90.8%) to heavy metal pollution in the water columns (Figure 4a). In contrast, other assessed heavy metals were at a safe concentration (Figure 4a). In addition, the Wilcox test showed that the $P_{n}$ of heavy metals and $P_{i}$ of Hg in surface water were significantly higher than those in bottom water (all *p* values < 0.05), and the $P_{n}$ and $P_{i}$ for Hg at most sites showed slight pollution in surface water (Figure 4b,c). The results indicate that the heavy metal pollution dominated by Hg has certain stratification in the water column.

The geo-accumulation index was used to evaluate heavy metal pollution in the sediments. The results showed that Cd and Pb (mean $I_{geo}$ was 0.72 and 0.007, respectively) had slight–extreme and slight–moderate pollution levels ($I_{geo}$ > 0), respectively; also, Cu, Zn, As, and Cr (all mean $I_{geo}$ values < 0) polluted to a lesser extent (Figure 4d). The $I_{geo}$ values for Cd and Pb in the sediments at the depth of 0–9 cm were significantly higher than that at 9–45 cm (Figure 4e,f). From the perspective of different lakes, slight pollution by Cd (mean $I_{geo}$ 0.50) was present in Honghu Lake; slight pollution by Cd, Cr, and Pb (mean $I_{geo}$ 0.26, 0.06, and 0.03, respectively) was present in East Lake; in Daye Lake, heavy pollution was caused by Cd (mean $I_{geo}$ was 3.73), Cu, As and Pb caused moderate pollution (mean $I_{geo}$ 1.38, 1.17, and 1.00, respectively), and Zn resulted in slight pollution (mean $I_{geo}$ 0.76); Cd and Pb resulted in slight pollution at a few Liangzi Lake sites. Similar heavy metal pollution has been observed in the Han River, which is also located in the Jianghan Plain [33]. These results indicate that Cd is the primary contributor to heavy metal pollution in the Jianghan Plain, followed by Pb.

### 3.4. Potential Ecological Risk Assessment of Heavy Metals

**Figure 5:**
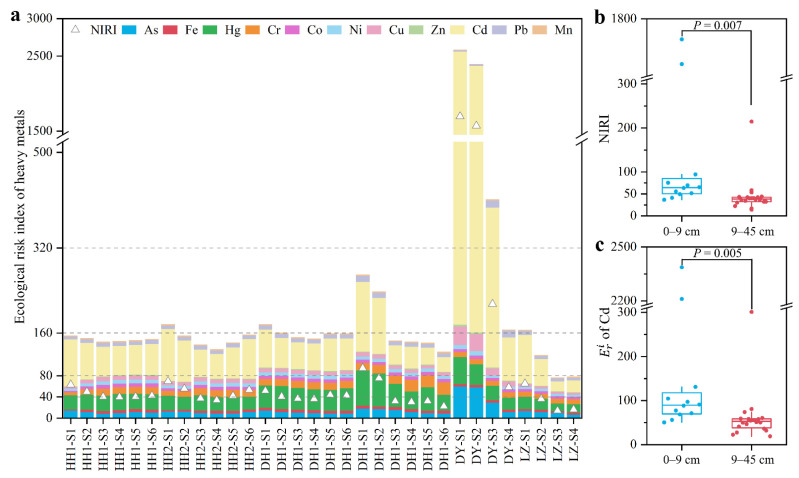
Potential ecological risk of heavy metals in sediment core: (**a**) NIRI and $E_{r}^{i}$ values of heavy metals; (**b**) NIRI difference in heavy metals within sediments at 0–9 and 9–45 cm; and (**c**) $E_{r}^{i}$ difference in Cd within sediments at 0–9 and 9–45 cm. The gray dotted line represents the threshold of different ecological risk levels of heavy metals: 40, 80, 160 and 320 represents moderate, considerable, high, and extremely high risk, respectively.

In this study, $E_{r}^{i}$ and NIRI were performed to assess the ecological risk of heavy metals in the sediments. The order of average value of $E_{r}^{i}$ was as follows: Cd (208.3) > Hg (33.1) > As (15.5) > Cr (12.8) > Cu (8.6) > Pb (8.0) > Ni (6.0) > Zn (1.48) > Mn (1.23) > Co (1.18) > Fe (0.96). As shown in Figure 5a, Cd had moderate–extreme ecological risks ($E_{r}^{i}$ > 80) in 84.4% of samples, whereas other heavy metals had almost low ecological risk ($E_{r}^{i}$ < 40). NIRI showed that heavy metals in 62.5% of samples had moderate to extremely high ecological risks (Figure 5a). The contribution rate of ecological risks of heavy metals can be measured by $E_{r}^{i}$ [33], and Cd was the primary contributor (mean is 43.4%) to the ecological risk. Similarly, Cd, as the heavy metal with the highest ecological risk, also occurred in the adjacent Han River [33]. These results suggest that the ecological risk of heavy metals mainly comes from Cd in the Jianghan Plain. The highest ecological risk of Cd may be related to the intensive chemical and electronics industry in this region, which is the crucial source of Cd [37,46]. In addition, the NIRI of heavy metal and $E_{r}^{i}$ of Cd in surface sediment (0–9 cm) were significantly higher than those in bottom sediment (9–45 cm) (all *p* values < 0.01; Figure 5b,c). These results indicate that not only the surface sediment has higher ecological risk of heavy metals than the bottom sediment, but also reveal that the higher ecological risk of heavy metals (especially Cd) occurred in recent years than that in the past decades. Similarly, the ecological risk of Cd is also dominant in the surface sediment of Dianchi Lake, Dongting Lake, and Poyang Lake [8,12,44]. A recent review also confirmed that Cd has become the heavy metal with the most serious ecological risk during the past 20 years in China [3].

### 3.5. Source Identification of Heavy Metals

**Figure 6:**
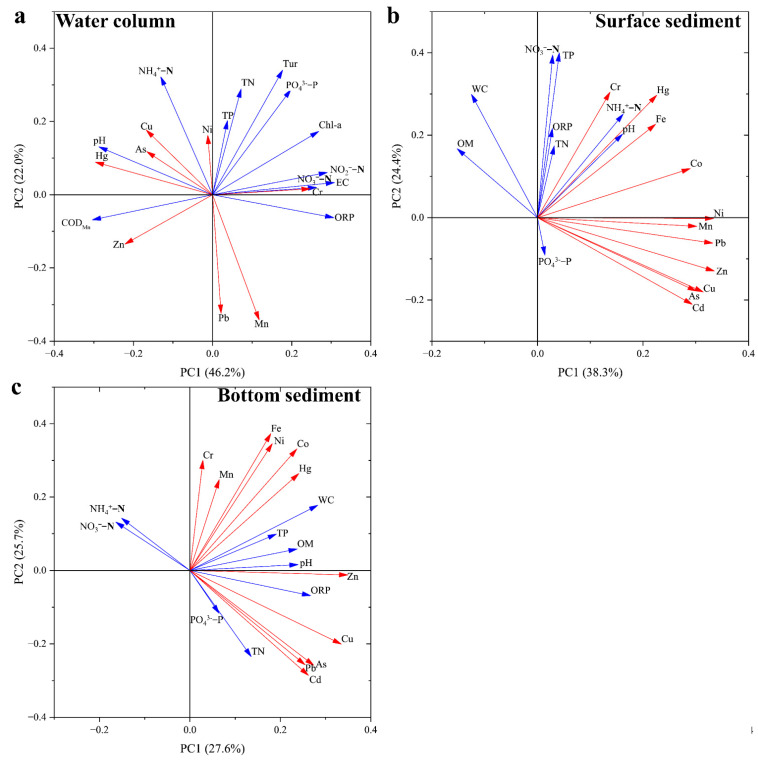
PCA of heavy metals and physiochemical parameters in the water column (**a**), surface sediment (**b**), and bottom sediment (**c**).

PCA was used to explore the potential sources of heavy metal pollution. Heavy metals in water represent the current pollution situation. For the water columns, the two principal components extracted explained 68.2% of the total variance (Figure 6a). Principal component 1 (PC1) is dominated by Hg, Cu, As, Zn, and Cr. A significant positive relationship exists in these heavy metals and physiochemical parameters, indicating that they may have common sources. Specifically, Hg, As, and COD_Mn_ are crucial components of pesticides [38], while NH_4_^+^-N is an important indicator of agricultural pollution [47]. Cr is the excreta of the electroplating industry, which may promote a rise in EC [48]. The accumulation of Cu and Zn in aquatic environments mainly comes from animal excreta, because these two metals are usually added to animal feed as growth promoters [49]. At present, Jianghan Plain has intensive rice planting, aquaculture, and chemical plants [13]. Therefore, PC1 can be regarded as indicating agricultural and chemical industry pollution. Principal component 2 (PC2) is dominated by Pb, Mn, and Ni. Ni is considered an indicator of fuel combustion [39,40] and used in industrial production as a raw material or catalyst [46,50]; thus, Ni and TN, TP, and NH_4_^+^-N lack the same source, although they have significant positive relationships. Pb is not only in the tail gas of oil combustion (entering the lake via atmospheric sedimentation) [51], but also an important element in automobile manufacturing (such as additives for airbag detonators) [52]; thus, automobile exhaust and industry are the major sources of Pb. Mn is commonly used as a reducing agent and catalyst in the production of materials such as alloys and magnetic components [53]. As a result, PC2 can be attributed to chemical industry and traffic pollution.

Surface sediments (0–9 cm) present heavy metal pollution during the last ~10 years. The two principal components extracted explain 62.7% of the total variance (Figure 6b). PC1 accounted for 38.3% of the total variation and is dominated by Co, Ni, Mn, Pb, Zn, Cu, As, and Cd. These heavy metals have either a single source or a shared source. For example, Pb is not only the waste from automobile manufacturing and fuel [51,52], but also an important component of feed additives together with Zn and Cu [49]. Apart from the production of fertilizer and pesticides (sodium arsenate and calcium arsenate) [38], As can also be used together with Co, Ni, and Mn in chemical industry and metal smelting as a combustion promoter or catalyst [39,51]. Cd and Zn are indispensable raw materials for brakes, tires, and lubricants [54]. Therefore, PC1 represents a mixed source of agriculture, transportation, and chemical industry. PC2 accounts for 24.4% of the total variation and is dominated by Cr, Hg, and Fe. Cr and Hg can enter the water environment through pesticides and herbicides [38,55], so the significant positive correlations among Hg, Cr, NH_4_^+^-N, TN, and TP indicate that Hg and Cr pollution came mainly from agriculture. Soil parent material is the primary contributor of Fe because the concentration of this element in surface sediments was almost lower than the background value (Figure 3h). Thus, PC2 can represent agriculture and the natural environment.

The heavy metal pollution in the bottom sediment (9–45 cm) can be traced to ~60 years ago based on previous investigations on the deposition rate of sediments in these lakes [34,35,36]. The two principal components extracted explain 53.3% of the total variance (Figure 6c). PC1 explains 27.6% of the total variation and is dominated by Zn, Cu, As, Pb, and Cd. These heavy metals (except Cd) are usually added to pesticides, fertilizers, or feedstuffs [38,49], and have significant positive correlations with agricultural nonpoint source pollutants (TN, TP, and PO_4_^3−^-P). Remarkably, the ratio of As concentration to the background value is 1.02, suggesting that the soil parent material is also an important source of As. These results imply that PC1 denotes agricultural pollution and the natural environment. PC1 explains 25.7% of the total variation and is dominated by Cr, Mn, Fe, Ni, Co, and Hg. In the past few decades, the steel-making industry has been regarded as the leading industry by the government [41], and Fe, Cr, Co, Ni, and Mn are used as raw materials, fuels, or catalysts for this industry [39,51]. Therefore, it is reasonable for PC2 to be viewed as reflecting the steel-making industry.

## 4. Conclusions

This study investigated the pollution, potential risks, and sources of 11 heavy metals in the shallow lake water columns and sediment cores. In the water columns, 10 heavy metals were detected and had insignificant stratification except for Hg. In the sediment core, 11 heavy metals were detected and they had three vertical profiles, namely, As, Hg, Cd, Pb, and Mn had higher concentrations in the surface sediment, and Cr, Co, Fe, and Ni had higher concentrations in the bottom sediment, while Cu and Zn had no significant stratification. In the water columns, heavy metals reached slight–moderate pollution levels and Hg was the primary contributor to the pollution. In the sediment cores, heavy metal pollution reached slight–extreme levels and was dominated by Cd and Pb. Risk assessments showed that heavy metals in sediments posed moderate–extreme ecological risks, and the ecological risk came mainly from the surface sediments and Cd. The source apportionment revealed that heavy metal pollution in water and surface sediments had similar sources, i.e., agriculture, transportation, and chemical industry; agriculture and steel-making were the primary pollutant sources in bottom sediments. This study sheds light on the pollution, risks, and sources of heavy metals in the vertical profiles of lakes within the densely populated plain, and provides scientific information for understanding the heavy metal residues in the vertical profile of lakes.

## Figures and Tables

**Table 1:** Classifications of heavy metal pollution and risk rank of $P_{i}$, $P_{n}$, $I_{geo,}$ $E_{r}^{i}$, and NIRI.

Class	$P_{i}$		$P_{n}$		$I_{geo}$		$E_{r}^{i}$		NIRI	
	Scope	Pollution Rank	Scope	Pollution Rank	Scope	Pollution Rank	Scope	Risk Rank	Scope	Risk Rank
1	≤1	Unpolluted	≤0.7	Unpolluted	≤0	Unpolluted	≤40	Low risk	≤40	Low risk
2	1–2	Slight pollution	0.7–1	Slight pollution	0–1	Slight pollution	40–80	Moderate risk	40–80	Moderate risk
3	2–3	Moderate pollution	1–2	Moderate pollution	1–2	Moderate pollution	80–160	Considerable risk	80–160	Considerable risk
4	≥3	Heavy pollution	≥2	Heavy pollution	2–3	Moderate to Heavy pollution	160–320	High risk	160–320	High risk
5					3–4	Heavy pollution	≥320	Extremely high risk	≥320	Extremely high risk
6					4–5	Heavy to extreme pollution				
7					≥5	Extreme pollution				
Reference	[22]		[24]		[25]		[22]		[26]	

**Table 2:** Comparison of heavy metals in Honghu Lake, Daye Lake, Liangzi Lake, and East Lake with other surface waters in China (μg/L).

	Dianchi Lake	Taihu Lake	Chaohu Lake	Dongting Lake	Caohai Lake	Yangtze River	Haihe River	Pearl River	Honghu Lake	Donghu Lake	Daye Lake	Liangzi Lake
As	2.78 ^a^		8.21	3.62 (1.77–6.91) ^b^	(1.45–2.97)	3.41	2.34 (nd–5.34)		0.46–1.83	1.37–4.03	15.70–33.37	0.45–0.56
Hg				nd	(0.03–0.14)	0.14	0.14 (nd–0.47)		0.06–0.10	0.08–0.14	0.12–0.14	0.11–0.13
Cr	1.54	1.29 (0.27–3.81)	0.50	0.62 (0.15–1.03)	(2.25–5.59)		28.18 (1.22–47.04)	8.5	nd–1.29	nd–0.35	nd–0.26	nd
Ni	2.05	2.44 (0.28–6.37)	26.47	1.51 (0.29–5.11)			20.33 (0.87–33.6)	12.5	0.51–1.52	nd–0.59	1.23–2.59	0.16–0.42
Cu	1.36	2.88 (0.96–6.24)	2.56	2.50 (0.70–7.65)	(1.83–2.63)	2.86	2.81 (1.37–8.35)	1.6	1.15–1.56	1.23–1.93	2.16–3.01	1.10–1.12
Zn	20.64	8.78 (2.49–18.52)	23.05	20.91 (2.81–71.24)	(28.92–55.78)	5.40	26.17 (0.30–196.05)	8.9	2.41–3.79	3.59–5.84	2.68–3.64	4.31–4.33
Cd	0.22	0.05 (0.03–0.08)	0.58	0.05 (nd–0.15)	(0.25–3.53)	0.97	0.06 (nd–0.63)	2.9	nd	nd	nd–0.06	nd
Pb	0.54		3.51	1.49 (nd–3.66)	(2.00–6.74)	4.69	0.45 (nd–1.46)	12.8	0.19–0.46	0.16–0.86	0.23–0.24	0.35–0.61
Mn	4.32	1.73 (0.01–7.53)					42.16 (4.14–188.67)		0.53–3.71	0.33–3.73	0.63–1.50	3.04–3.42
Reference	[12]	[7]	[11]	[8]	[30]	[31]	[4]	[32]	This study	This study	This study	This study

Note: ^a^ average concentration; ^b^ concentration range.

**Table 3:** Comparison of heavy metals in sediment cores from Honghu Lake, Daye Lake, Liangzi Lake, and East Lake with other surface sediments in China (mg/kg, dry weight).

	Dianchi Lake	Taihu Lake	Chaohu Lake	Dongting Lake	Poyang Lake	Yangtze River	Haihe River	Pearl River	Honghu Lake	Donghu Lake	Daye Lake	Liangzi Lake
As	2.06 ^a^			29.22 (16.04–64.28) ^b^	(2.2–30.3)	20.1 (8.9–33.9)	1.35 (nd–7.65)	21.99 (3.34–37.11)	14.5 (11.5–18.8)	16.2 (11.7–24.0)	51.5 (15.6–77.1)	14.0 (9.79–17.4)
Hg				0.18 (0.05–0.47)		0.03 (0.01–0.09)	0.07 (nd–0.84)	0.13 (0.01–0.25)	0.07 (0.06–0.08)	0.12 (0.08–0.17)	0.09 (0.06–0.13)	0.05 (0.04–0.06)
Fe	50,720				(6200–56,000)				48,614 (24,510–59,358)	55,335 (51,718–57,385)	46,376 (44,295–48,420)	40,891 (39,746–41,704)
Cr	74.78	138.4 (9.35–464.9)	61.0 (28.7–91.1)	89.0 (53.5–116.0)	(5.8–88.4)		28.35 (9.31–73.23)	78.4 (12–130)	100.3 (47.1–119)	138.3 (103–200)	82.6 (78.9–85.9)	79.2 (78.2–80.2)
Ni	45.81	47.9 (11.5–114.9)	36.0 (14.8–59.1)	41.7 (23.0–54.9)	(2.7–50.2)	31.5 (26.1–33.9)	15.42 (4.73–23.96)		49.5 (27.4–55.2)	50.9 (46.1–54.8)	47.8 (37.6–61.0)	31.9 (29.7–34.1)
Cu	146.2	35.1 (11.8–134.6)	26.9 (12.6–41.8)	45.5 (34.9–73.4)	(2.7–245.9)	28.5 (13.9–37.0)	12.26 (2.52–26.20)	46.8 (5.8–170.6)	44.4 (24.0–50.9)	44.7 (37.7–51.1)	143.1 (53.1–224)	29.4 (25.6–33.7)
Zn	496.8	89.7 (16.7–295.9)	341 (1.5–907)	322.6 (227.0–463.4)	(13.3–311.8)	104.1 (71.9–130.9)	46.0 (10.1–82.9)	143.1 (32–259)	116.9 (69.3–130)	127.1 (116–138)	237.5 (151–319)	76.2 (65.2–94.5)
Cd	13.2	1.35 (0.03–4.09)	17.5 (0.04–42.4)	2.87 (0.66–7.89)	(0.04–6.3)	0.67 (0.33–0.89)	0.11 (0.02–0.35)	0.46 (0.06–2.06)	0.33 (0.26–0.49)	0.30 (0.14–0.65)	6.2 (0.40–11.9)	0.23 (0.09–0.45)
Pb	108.8	38.3 (0.01–93.6)	47.5 (1.56–113)	58.0 (39.0–102.9)	(15.5–71.8)	27.3 (16.9–41.8)	6.87 (1.4–34.8)	49.6 (23–78)	34.9 (28.6–37.5)	41.7 (36.7–60.0)	81.8 (67.7–96.5)	31.4 (26.2–40.8)
Mn	813	696.7 (116–1955)	482 (168–887)		(177–1656)	977 (725–1620)	229.2 (55.9–346.5)		934 (540–1318)	988 (707–1523)	1057 (537–1644)	642 (583–721)
Co	15.24		10.8 (4.4–16.7)		(2.0–24.8)		5.56 (1.87–9.2)		18.7 (9.4–21.9)	20.4 (19.7–21.4)	19.1 (17.9–21.0)	15.5 (14.9–16.0)
Reference	[12]	[7]	[11]	[8]	[44]	[31]	[4]	[45]	This study	This study	This study	This study

Note: ^a^ average concentration; ^b^ concentration range.

## Data Availability

Data are provided in Supplementary Materials.

## Supplementary Materials

The following supporting information can be downloaded at: https://www.mdpi.com/article/10.3390/ijerph20043676/s1, Figure S1: Differences of heavy metals in different lake sediments; Table S1: Physiochemical parameters of water; Table S2: Physiochemical parameters of sediments; Table S3: Reference value of heavy metal in Environmental Quality Standards for Surface Water (mg/L); Table S4: Environmental background values of heavy metals in Hubei province (mg/kg); Table S5: Table S5 Concentration of heavy metals in water column (μg/L); Table S6: Concentration of heavy metals in sediment cores (mg/kg).
