# Supplementary material for: Occurrence, Risk, and Source of Heavy Metals in Lake Water Columns and Sediment Cores in Jianghan Plain, Central China
Source: Int J Environ Res Public Health. 2023 Feb 19;20(4):3676. doi: 10.3390/ijerph20043676 (PMC9963068; doi:10.3390/ijerph20043676)
Supplement: Supplementary file 1 [file ijerph-20-03676-s001.zip › ijerph-2195975-supplementary - final.pdf]

Supplementary material

Table S1 Physiochemical parameters of water.

Site	Depth	TN (mg/L)	NH_4^+-N (mg/L)	NO_3^--N (mg/L)	NO_2^--N (mg/L)	TP (mg/L)	PO_4^{3-}-P (mg/L)	COD_{Mn} (mg/L)	WT (°C)	Do (mg/L)	ORP (mv)	pH	Chl-a (μg/L)	Tur (FTU)	EC (μS/cm)
HH1	Surface	1.30	0.33	0.05	0.004	0.09	0.03	18.75	28.67	11.73	−4.48	9.09	23.14	12.48	306.6
	Bottom	1.07	0.66	0.10	0.003	0.12	0.01	19.08	28.22	9.54	4.41	8.88	29.46	14.35	306.0
HH2	Surface	0.95	0.15	0.50	0.060	0.19	0.05	13.75	29.51	10.54	8.91	8.63	29.32	13.90	414.0
	Bottom	0.83	0.07	0.53	0.066	0.13	0.02	13.08	29.01	8.92	11.41	8.55	22.05	15.06	412.6
LZ	Surface	0.65	0.04	0.12	0.004	0.12	0.01	18.75	29.99	7.75	−10.66	8.37	16.98	6.74	386.1
	Bottom	0.73	0.10	0.12	0.004	0.13	0.01	19.08	29.59	7.34	−9.42	8.18	19.91	7.70	388.0
DY	Surface	0.78	0.17	0.11	0.003	0.09	0.01	21.75	32.24	10.51	−35.44	9.27	8.37	11.24	186.0
	Bottom	0.72	0.18	0.13	0.002	0.08	0.01	23.08	32.09	10.00	−20.85	9.15	8.95	11.89	192.5
DH1	Surface	0.97	0.65	0.20	0.002	0.25	0.02	22.58	32.13	11.52	−14.21	9.24	12.09	10.58	243.5
	Bottom	1.00	0.06	0.24	0.004	0.24	0.04	21.92	31.20	9.46	−14.44	9.13	14.63	11.87	194.3
DH2	Surface	0.65	0.10	0.18	0.002	0.12	0.00	21.42	29.92	8.91	−6.24	9.03	9.93	5.41	266.2
	Bottom	0.74	0.08	0.17	0.002	0.10	0.00	22.42	29.14	7.86	−6.50	8.83	9.36	5.60	252.4

Table S2 Physiochemical parameters of sediments.

Site	Depth (cm)	TN (μg/g)	NH_4^+-N (μg/g)	NO_3^--N (μg/g)	TP (μg/g)	PO_4^{3-}-P (μg/g)	WC	OM	pH	ORP (mv)
HH1	0–3	3446.1	19.6	3.9	368.5	0.97	67.1%	14.6%	6.82	-68
	3–6	4109.2	16.8	5.0	393.7	1.26	56.3%	9.8%	6.50	−134
	6–9	2173.3	12.4	4.3	412.5	0.68	51.2%	8.4%	6.32	−116
	9–12	2042.9	14.5	5.5	901.3	0.76	48.6%	7.7%	6.54	−175
	12–15	500.3	30.5	11.9	569.3	0.91	46.5%	6.5%	6.41	−235
	15–18	1672.6	44.9	18.0	340.0	1.44	44.7%	6.3%	6.49	−253
	18–21	543.1	92.0	37.7	602.8	1.08	43.5%	7.0%	6.40	−228
	21–24	1230.5	123.4	51.8	502.0	0.80	38.5%	6.5%	6.24	−261
	24–27	1081.8	111.9	48.1	499.1	1.25	39.5%	6.6%	6.29	−240
	27–30	926.0	188.0	82.7	592.9	0.99	42.6%	6.7%	6.26	−229
	30–33	1018.0	212.8	95.7	390.3	0.87	39.8%	6.3%	6.13	−272
	33–36	802.1	271.4	124.8	446.7	1.40	40.5%	6.5%	6.18	−234
	36–39	999.1	261.2	122.8	327.3	0.82	40.3%	6.8%	6.10	−241
	39–42	706.3	269.0	129.1	427.5	1.24	39.5%	6.7%	5.91	−258
	42–45	1217.3	258.5	126.7	951.7	1.33	40.1%	6.8%	5.97	−299
HH2	0–3	5145.8	25.2	5.0	549.7	1.95	62.7%	10.5%	6.96	−78
	3–6	4599.2	21.2	6.4	589.5	1.80	53.2%	10.1%	6.68	−148
	6–9	4151.2	10.5	3.7	411.1	1.48	50.7%	9.8%	6.56	−166
	9–12	1006.5	15.5	5.9	507.4	0.80	42.1%	6.7%	6.58	−181
	12–15	856.8	4.6	1.8	457.7	1.40	41.1%	6.3%	6.32	−188
	15–18	977.9	9.1	3.7	438.4	2.26	39.1%	6.1%	6.39	−198
	18–21	1430.1	52.3	21.4	306.9	1.80	41.0%	6.6%	6.29	−208
	21–24	1653.4	46.4	19.5	410.8	0.96	39.4%	6.6%	6.21	−224
	24–27	2233.2	163.0	70.1	428.5	1.34	38.7%	6.8%	6.13	−216
	27–30	2609.5	67.1	29.5	379.9	1.71	44.0%	7.4%	6.09	−271
	30–33	3974.4	120.8	54.4	298.4	1.86	43.2%	7.2%	6.14	−243

	33–36	4124.5	60.9	28.0	301.7	0.95	42.2%	7.4%	5.95	−284
	36–39	3839.8	109.1	51.3	312.5	0.97	43.8%	7.6%	5.98	−251
	39–42	7595.4	119.8	57.5	556.4	2.72	41.8%	7.3%	5.79	−301
	42–45	5885.9	104.0	50.9	464.7	1.55	40.7%	7.6%	5.85	−270
LZ	0–3	3939.1	13.6	2.7	210.9	3.73	57.1%	9.8%	6.84	−89
	3–6	3161.2	4.4	1.3	225.8	2.79	51.3%	8.4%	6.51	−178
	6–9	2628.0	10.6	3.7	303.8	3.50	47.9%	8.2%	6.39	−206
	9–12	1793.9	17.2	6.9	373.3	2.63	43.2%	7.0%	6.29	−241
	12–15	1718.4	3.8	1.7	252.8	0.97	39.2%	6.7%	6.41	−232
	15–18	1503.7	63.9	30.0	403.1	5.47	38.2%	6.5%	6.26	−256
	18–21	1603.0	95.2	47.6	361.8	2.17	34.2%	6.2%	6.21	−245
	21–24	701.9	75.3	47.5	226.0	2.32	31.1%	5.9%	6.08	−288
	24–27	897.0	57.8	37.6	339.4	2.59	29.9%	6.0%	6.16	−271
DY	0–3	3702.5	4.2	0.8	493.5	1.83	51.2%	9.5%	7.04	−72
	3–6	3646.5	8.2	2.5	476.7	1.77	50.4%	8.1%	6.83	−133
	6–9	3525.4	21.2	7.4	362.6	1.89	48.8%	7.6%	6.79	−161
	9–12	3135.0	26.5	10.6	319.7	1.74	45.1%	7.5%	6.52	−218
	12–15	2783.1	5.4	2.4	217.0	1.61	43.6%	7.2%	6.41	−243
	15–18	2270.4	21.4	10.1	870.9	2.44	42.3%	7.5%	6.54	−232
	18–21	2699.1	14.4	7.2	533.2	2.27	39.2%	7.8%	6.32	−251
	21–24	2320.1	172.3	108.5	212.0	2.07	36.6%	7.4%	6.18	−265
	24–27	1795.8	72.8	47.3	254.7	2.10	32.6%	7.6%	6.13	−309
DH1	0–3	5427.6	4.7	1.4	708.9	4.06	58.8%	10.5%	7.05	−45
	3–6	3022.4	4.1	1.6	680.0	3.02	51.1%	8.5%	6.76	−154
	6–9	2056.8	2.1	1.2	479.0	2.72	47.3%	8.2%	6.68	−176
	9–12	2281.0	27.0	14.8	533.7	2.78	48.2%	8.3%	6.71	−208
	12–15	1739.2	48.2	26.5	355.8	3.40	46.4%	8.1%	6.59	−236
	15–18	2054.8	60.0	33.0	370.0	5.10	44.9%	7.8%	6.62	−234

	18–21	1936.2	75.4	41.4	362.6	3.72	46.2%	7.4%	6.42	−227
	21–24	1578.6	117.8	64.7	490.7	3.40	46.0%	7.2%	6.24	−246
	24–27	1131.5	128.1	70.4	622.4	4.04	45.1%	6.8%	6.27	−241
	27–30	1659.7	131.4	72.2	494.0	1.74	46.2%	7.9%	6.37	−289
	30–33	1374.8	96.9	53.2	875.1	1.86	45.4%	9.3%	6.43	−231
	33–36	1523.2	25.8	14.2	422.9	2.02	44.4%	9.0%	6.40	−242
	36–39	1491.3	113.6	62.4	372.0	0.25	43.0%	7.9%	6.28	−253
	39–42	1698.0	175.2	96.2	619.0	0.74	42.8%	6.7%	6.18	−284
	42–45	1274.9	154.5	84.8	397.6	2.93	42.1%	6.2%	6.20	−261
DH2	0–3	5967.4	3.7	1.1	716.9	0.08	66.6%	10.4%	6.99	−64
	3–6	4595.9	59.6	20.9	525.6	0.27	63.1%	13.8%	6.75	−123
	6–9	4760.1	41.1	22.6	392.7	0.04	59.2%	12.9%	6.69	−149
	9–12	4008.1	12.4	6.8	349.2	0.11	56.8%	9.9%	6.58	−218
	12–15	2783.7	31.1	17.1	367.7	0.23	54.1%	9.3%	6.49	−247
	15–18	2723.1	76.8	42.2	286.6	0.11	52.3%	8.5%	6.52	−228
	18–21	2788.4	88.8	48.8	380.9	0.25	49.5%	8.2%	6.58	−231
	21–24	1909.1	102.9	56.5	409.3	0.26	47.1%	7.9%	6.52	−254
	24–27	2127.1	131.3	72.1	292.3	0.36	45.1%	7.7%	6.55	−238
	27–30	1939.3	125.8	69.1	320.6	0.41	43.7%	7.4%	6.43	−231
	30–33	1053.7	153.1	84.1	464.9	0.25	42.5%	7.3%	6.24	−243
	33–36	921.4	264.8	145.4	378.7	1.23	41.1%	7.3%	6.23	−261
	36–39	1656.1	171.1	94.0	374.8	0.37	40.7%	7.1%	6.25	−254
	39–42	1345.7	92.9	51.0	370.2	0.07	39.8%	6.9%	6.07	−260
	42–45	1399.5	170.3	93.5	280.3	0.33	38.8%	6.7%	6.01	−296

Table S3 Reference value of heavy metal in Environmental Quality Standards for Surface Water (mg/L).

	Cu	Zn	As	Hg	Cd	Pb	Cr
Reference value	1	1	0.05	0.0001	0.005	0.05	0.05

Table S4 Environmental background values of heavy metals in Hubei province (mg/kg).

	As	Hg	Fe	Cr	Co	Ni	Cu	Zn	Cd	Pb	Mn
Background value	12.8	0.079	52050	85.9	16.1	39.5	31.8	88.1	0.15	26.9	755

Table S5 Concentration of heavy metals in water column (μg/L).

Site	Depth	As	Hg	Fe	Cr	Co	Ni	Cu	Zn	Cd	Pb	Mn
HH1	Top	0.46	0.10	0.00	0.38	<0.01	0.89	1.19	3.79	0.00	0.29	1.31
	Bottom	0.63	0.08	0.00	0.00	<0.01	0.51	1.15	2.41	0.00	0.19	0.53
HH2	Top	1.83	0.06	0.00	0.54	<0.01	1.12	1.56	3.05	0.00	0.46	3.71
	Bottom	1.48	0.06	0.00	1.29	<0.01	1.52	1.46	3.32	0.00	0.28	1.76
DY	Top	15.70	0.12	0.00	0.00	<0.01	1.23	2.16	2.68	0.00	0.23	0.63
	Bottom	33.37	0.14	0.00	0.26	<0.01	2.59	3.01	3.64	0.06	0.24	1.50
LZ	Top	0.56	0.13	46.50	0.00	<0.01	0.16	1.10	4.33	0.00	0.35	3.42
	Bottom	0.45	0.11	0.00	0.00	<0.01	0.42	1.12	4.31	0.00	0.61	3.04
DH1	Top	4.03	0.14	0.00	0.24	<0.01	0.56	1.93	4.04	0.00	0.16	0.33
	Bottom	3.88	0.08	0.00	0.00	<0.01	0.00	1.40	5.84	0.00	0.86	3.28
DH2	Top	1.85	0.12	0.00	0.25	<0.01	0.45	1.49	4.54	0.00	0.81	3.13
	Bottom	1.37	0.08	0.00	0.35	<0.01	0.59	1.23	3.59	0.00	0.34	3.73

Table S6 Concentration of heavy metals in sediment cores (mg/kg).

Site	Depth	As	Hg	Fe	Cr	Co	Ni	Cu	Zn	Cd	Pb	Mn
HH1	HH1-S1	18.8	0.071	24510.5	47.1	9.4	27.4	24.0	69.3	0.44	28.6	540.0
	HH1-S2	15.7	0.074	45104.3	88.2	18.4	43.3	40.0	98.8	0.34	35.1	954.4
	HH1-S3	11.5	0.076	55024.0	111.3	21.9	54.8	50.9	127.0	0.28	36.6	1023.2
	HH1-S4	13.5	0.080	54247.5	112.0	21.3	55.0	48.2	123.3	0.27	35.3	928.4
	HH1-S5	15.5	0.078	55148.9	110.4	21.2	54.9	48.7	128.5	0.28	35.8	968.6
	HH1-S6	14.4	0.078	54764.6	113.8	21.5	55.2	47.7	129.6	0.29	36.5	973.1
HH2	HH2-S1	16.3	0.070	37230.9	81.9	15.4	44.5	44.5	120.1	0.49	32.8	1318.4
	HH2-S2	16.6	0.064	40757.0	85.1	16.2	43.7	42.4	105.8	0.39	32.9	1040.0
	HH2-S3	12.9	0.075	53099.5	111.2	20.8	54.2	47.6	122.7	0.26	35.9	1000.2
	HH2-S4	11.7	0.066	59358.1	112.7	20.3	54.2	46.1	121.8	0.23	34.5	1315.2
	HH2-S5	12.1	0.068	52999.3	119.0	20.1	54.8	45.9	128.0	0.29	37.5	604.8

	HH2-S6	15.7	0.062	51125.7	110.3	18.6	52.6	47.1	127.4	0.37	37.3	545.1
DY	DY-S1	77.1	0.134	46907.7	78.9	19.5	54.3	224.0	318.5	11.93	92.8	1643.6
	DY-S2	74.8	0.103	48420.2	85.8	21.0	61.0	201.9	318.5	11.05	96.5	1288.7
	DY-S3	38.6	0.072	44295.3	79.9	17.9	37.6	83.2	161.8	1.50	70.2	757.7
	DY-S4	15.6	0.060	45880.0	85.9	18.1	38.1	63.1	151.2	0.40	67.7	536.6
LZ	LZ-S1	17.4	0.062	41704.5	80.2	15.7	34.1	33.7	94.5	0.45	40.8	582.9
	LZ-S2	15.9	0.055	41307.4	79.8	16.0	32.9	30.8	78.8	0.25	31.3	589.7
	LZ-S3	13.0	0.038	40808.7	78.2	15.5	30.9	27.3	66.2	0.09	27.2	720.7
	LZ-S4	9.8	0.041	39746.3	78.5	14.9	29.7	25.6	65.2	0.11	26.2	674.2
DH1	DH1-S1	18.9	0.111	55467.1	102.7	19.8	48.9	49.1	137.5	0.36	43.1	1054.7
	DH1-S2	15.7	0.115	55164.7	112.0	20.3	51.6	45.3	125.0	0.28	38.5	990.9
	DH1-S3	14.5	0.108	56272.1	116.7	20.5	53.5	47.1	125.2	0.25	35.5	1123.6
	DH1-S4	13.3	0.103	54739.5	117.3	20.6	54.8	47.7	131.7	0.25	38.5	828.7
	DH1-S5	11.7	0.103	56000.1	116.9	21.4	54.7	44.6	136.4	0.30	41.4	777.3
	DH1-S6	12.1	0.111	54015.9	120.4	20.0	52.3	41.0	126.9	0.29	39.7	707.3
DH2	DH2-S1	24.0	0.174	54632.2	120.6	20.4	46.2	51.1	138.5	0.65	60.0	1110.3
	DH2-S2	22.3	0.164	55858.8	133.1	19.7	46.1	49.6	132.2	0.52	54.6	849.3
	DH2-S3	20.9	0.114	57384.7	132.9	20.3	51.9	42.9	116.2	0.18	36.7	1081.9
	DH2-S4	15.7	0.088	55817.3	189.1	20.9	52.8	41.7	122.4	0.20	37.0	1021.2
	DH2-S5	12.7	0.115	51717.5	197.8	20.6	49.1	37.7	115.9	0.16	36.8	781.3
	DH2-S6	12.4	0.078	56946.9	200.5	20.0	48.4	39.1	117.2	0.14	38.6	1523.3

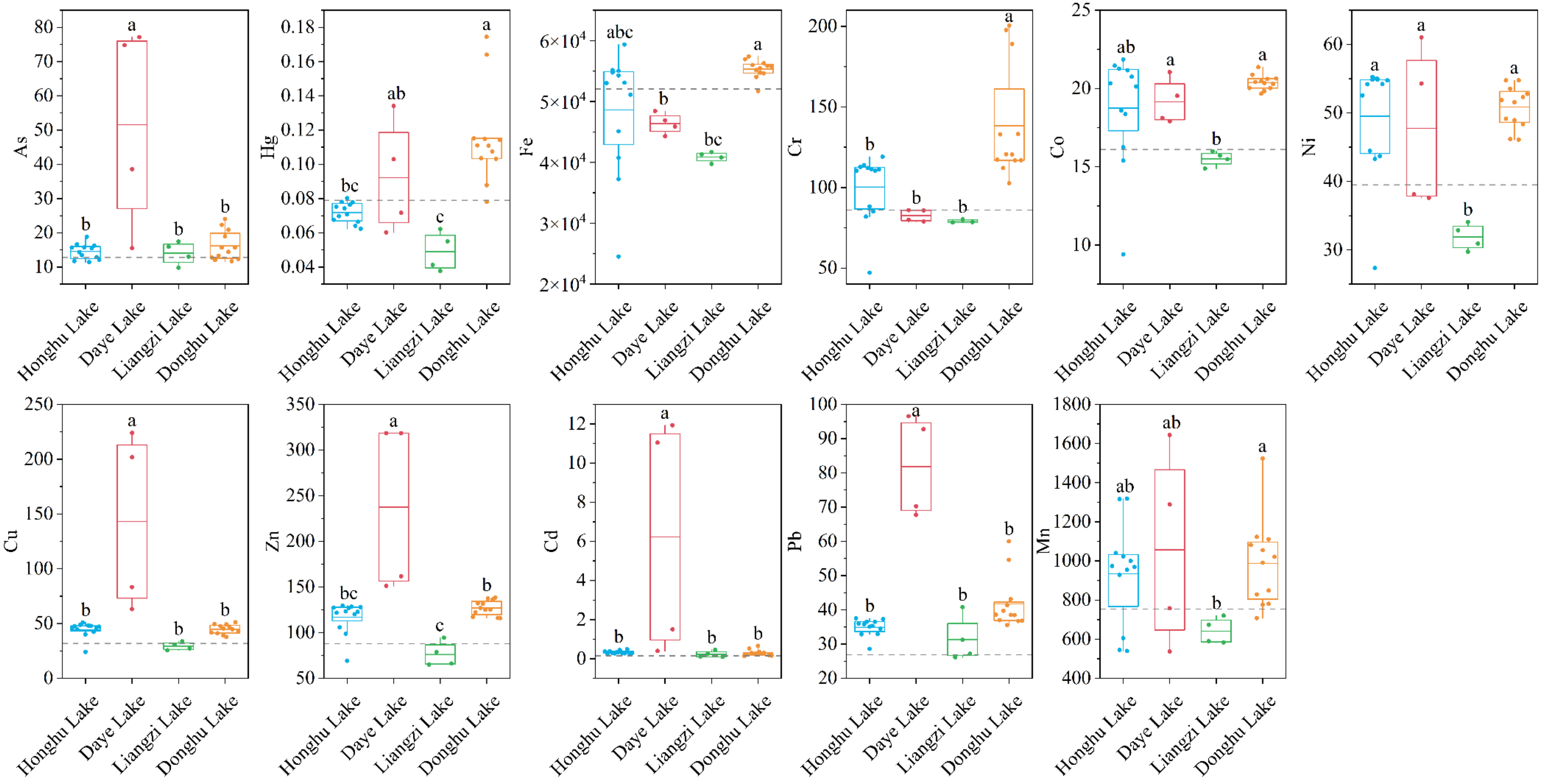

Figure S1 Differences of heavy metals in different lake sediments. Different letters (a, b, or c) represent statistically significant differences at the $p < 0.05$ level.
